# Hope and Fulfillment After Complex Trauma: Using Mixed Methods to Understand Healing

**DOI:** 10.3389/fpsyg.2019.02061

**Published:** 2019-09-20

**Authors:** Denise Saint Arnault, Laura Sinko

**Affiliations:** Department of Health Behavior and Biological Sciences, University of Michigan School of Nursing, Ann Arbor, MI, United States

**Keywords:** gender based violence, trauma recovery, childhood sexual abuse, positive psychology, emotional health, well-being

## Abstract

Research has shown that the experience of Childhood Sexual Abuse (CSA) can increase the rates of physical and emotional sequela, including depression, anxiety, PTSD, and physical pain. However, little is known about the healing journeys for those women who, after surviving CSA, also experience unwanted sexual experiences (USE) as a young adult. The goal of this mixed method study is to compare identity, distress, and positive health outcomes for survivors of CSA and USE with those of women who have survived USE alone. First, 206 women completed a survey that evaluated mental health distress, trauma centrality, and health indicators, and these women were invited to take part in an additional interview. A subsample of 24 women agreed to be interviewed with a narrative interview that examined trauma recovery from a holistic perspective. Thematic analysis of the interview data revealed contextual and internal barriers and facilitators of healing. Eight qualitative dimensions were derived from code frequencies of the emerging 50 qualitative codes, including Normalization, Denial, Negative Impact, Positive Coping, Rumination, Identity Healing, Hope, and Fulfillment Healing, and Engagement Healing. We then used *t*-tests to compare the mean code frequencies of these code complexes for survivors of CSA/USE and USE alone, and the significant findings revealed hypotheses to be tested in the larger quantitative sample. H1: Survivors of CSA will have less denial than USE alone; H2: Survivors of CSA will have higher Negative Impact scores than USE alone, and H3: Survivors of CSA will have more Hope and Fulfillment than USE alone. H1 was supported; survivors of both CSA and USE had a significantly higher mean Centrality Scores than Survivors of USE alone. H2 was supported, and all negative impact scores were significantly higher for the survivors of both CSA and USE than survivors of USE alone. H3 was partially supported. Survivors of both CSA and USE had significantly higher mean Post-Traumatic growth scores. However, survivors of USE alone had significantly higher means for Sense of Coherence and Self-Compassion scores. Implications for practice are discussed.

## Introduction

The immediate and long-term consequences of children’s exposure to maltreatment and other traumatic experiences are multifaceted. However, the compounded sequelae from exposures to both childhood sexual assault and sexual assault later in life make the trauma recovery process tremendously challenging. Researchers have defined “complex trauma” as multiple traumatic experiences which occur in childhood and adolescence, including multiple occurrences of emotional abuse and neglect, sexual abuse, and physical abuse (Ford et al., 2009; Kezelman and Stavropoulos, 2012; Cook et al., 2017). Complex trauma exposure results in a loss of core capacities for emotional self-regulation and interpersonal relatedness, and may also result in higher levels of cumulative impairment in mental and behavioral functioning, including psychiatric and addictive disorders, legal, vocational, and family problems, as well as physical illnesses (Cook et al., 2017).

One subset of women receiving national attention for their risk for sexual assault is college-aged women ages 18–24 years. College-aged women who are in college have twice the rates of sexual assault compared with all other survivors, and college-aged women who are not in college have three times these rates (Azimi and Daigle, 2017). This group is of critical importance from a developmental point of view because they are in the complicated transition from childhood to adulthood. While researchers in the past defined adolescence as 12 to 18 years of age (which corresponds to pubertal onset), more researchers are beginning to investigate the interactions between biological and contextual factors, such as higher risks, emotional reactivity, changes in the school and social environments, spending more time with peers, and increases autonomy that occur as a person matures into adulthood. In light of this clarity, recent work has expanded the definition and timeframe of adolescence to include young adulthood up to about 25 years of age. This timeframe encompasses neural changes that occur beyond eighteen years of age as well as behavioral and social demands and roles of young adulthood (Jaworska and MacQueen, 2015).

Research has shown that college-aged women are at higher risk of sexual assault; however, it is difficult to find statistics of rates of CSA in survivors of unwanted sexual experiences (USE) for college-aged women specifically. One issue that makes it difficult to assess the prevalence of childhood sexual abuse (CSA) and complex trauma is that definitions of sexual assault and CSA vary across studies (Murray et al., 2014). The WHO 2002 World Report on Violence and Health (2002) suggested that using only cases reported to authorities may hide the true magnitude of CSA or sexual violence (Krug et al., 2002). A systematic review of 39 prevalence studies in 21 countries found that the prevalence of CSA ranged from 0 to 53% for women and 0 to 60% for men. The investigators reported a combined mean prevalence of CSA in 7.9% of males and 19.7% of females, with the highest rates occurring in Africa and the lowest in Europe (Pereda et al., 2009). A similar definitional problem occurs when trying to capture the prevalence of sexual assault. For example, one systematical review of thirty-four studies on campus sexual assault reported the use of broad definitions of “sexual assault,” “rape,” unwanted sexual contact, and sexual coercion. These studies typically presented findings on a combination of two or more of these sexual victimization experiences, labeling them as sexual assault among college students, and did not provide breakdowns of the different forms of victimization (Fedina et al., 2018).

Despite these limitations, some research on complex trauma is available. Surveys of college women have found that between 15 and 20% have experienced rape by a man or men during their adolescence or adulthood (Schwartz and Leggett, 1999; Kahn et al., 2003; Littleton et al., 2009a, b). Another study combined report prevalence rates, citing rates ranging from 6 to 44.2% of women (Fedina et al., 2018). In a community sample of 117 adult rape victims assessed within 1 month of a recent rape showed that a history of child sexual abuse seems to increase vulnerability for adult sexual and physical victimization, and contribute to current PTSD symptoms within the cumulative context of other adult trauma (Nishith et al., 2000). Another study by Hannan et al. (2017) found that individuals with a history of CSA are more likely to experience both revictimizations in adolescence and PTSD symptoms in adulthood. Two studies found that 30% of adolescent/adult sexual assault victims experienced a newly completed rape over a follow-up period (Messman-Moore et al., 2005; Littleton et al., 2009a).

The phenomenon of revictimization is of particular importance for women who have experienced CSA. The literature consistently demonstrates evidence that child sexual abuse survivors are at greater risk of victimization later in life than the general population (Ports et al., 2016). However, there is variability in the prevalence rates of revictimization. A recent meta-analysis of 80 studies containing 12,252 survivors of child sexual abuse found the mean prevalence of sexual revictimization across studies was 47.9%, suggesting that almost half of child sexual abuse survivors are sexually victimized in the future (Walker et al., 2019). Another systematic review of 25 studies identified specific risk factors for revictimization included risky sexual behavior (particularly in adolescence), post-traumatic stress disorder, emotion dysregulation, and maladaptive coping. The only protective factor revealed in this study was perceived parental care (Scoglio et al., 2019).

Taken together, this literature documents the vulnerability, risk, and severe sequelae for people exposed to complex trauma in childhood and adolescence (including late adolescence up to age 24). However, one compelling issue that is neglected in this literature is the healing experiences of those vulnerable women who have experienced complex trauma. Researchers and practitioners need to understand not only incidence and prevalence of trauma and mental illness, but also resilience and recovery experiences from these horrific events. In a systematic review of over 37 articles on recovery after a sexual assault, the percentage of CSA survivors who were found to have normal levels of functioning despite a history of sexual abuse ranged from 10 to 53% (Domhardt et al., 2015). In the 37 studies included in this review, 10 to 53% of the CSA survivors self-reported a normal level of functioning despite a history of sexual abuse. Normal functioning was associated with a variety of protective factors. Protective factors included availability and use of supports from family and the broader social environment as well as internal attributes such as interpersonal and emotional competence, perceived emotional control, active coping, feelings of optimism, and external attribution of blame. These findings suggest that the study or health and resilience factors are an important scientific area to expand.

### The Rationale for Using Mixed Methods

As we have seen, there is limited research on trauma recovery for survivors of both CSA and USE. One characteristic of this limitation can be viewed as a methodological one, which includes the combined issues of self-definition, definitions of trauma, and definitions of healing. There have been qualitative studies illuminating healing processes (Herman, 1997; Saint Arnault and O’Halloran, 2014, 2016; Sinko et al., 2019; Sinko and Saint Arnault, in press). This research describes healing and recovery experiences as generally including managing memories and emotions, integration of experiences into one’s self-definition, connecting with others and connecting with that which is larger than oneself, such as spirit, nature, or God. Listening to survivors gives us a view into what they believe the dynamics of the recovery process is, and from these reports, we can derive theoretical propositions. From a mixed method point of view, we can use this qualitative data to test these propositions about recovery for a larger sample. Using mixed methods enhances our understanding of these relationships among the interview respondents by testing hypotheses in the same study.

### Aims and Hypotheses

This study aims to compare healing experiences for a sample of sexual assault survivors who did, and did not, also experience of childhood sexual assault. To do this, we use mixed methods to leverage qualitative and quantitative data. Using qualitative code frequencies of descriptions of healing journeys, we compare mean code frequencies to derive theoretical propositions about possible recovery dimensions. Next, we examined these recovery dimensions in the larger survey sample. Based on the analysis of mean frequencies of codes generated in from qualitative data analysis, we developed and will test the following hypotheses: (1) Survivors of CSA will have less minimization than survivors of USE alone; (2) Survivors of CSA will have more Negative Impact than survivors of USE alone; and (3) Survivors of CSA will have more hope and fulfillment than survivors of USE alone. We hope this project can begin to connect disparate literature by listening to survivor’s voices about healing, as well as examining trends in a larger sample to inform practice.

## Materials and Methods

### Design

This study compared trauma recovery dimensions for samples of sexual assault survivors who reported that they had also experienced childhood sexual assault with those who reported USE on the college campus. This study used an exploratory mixed method design that employed thematic analysis of qualitative interview data from a nested subsample to understand healing and trauma recovery. We then developed and tested the newly derived hypotheses in the larger quantitative dataset.

### Instruments and Measures

#### Trauma Impact

We used mental health symptomology scores to operationalize Trauma Impact. Mental health symptoms consisted of post-traumatic stress disorder, depression, anxiety, and somatic symptoms. *Post-traumatic Stress Disorder symptoms* were assessed with the PTSD Checklist for the DSM V (PCL-5) (Weathers et al., 2013). Response options are on a 5-point Likert scale (anchors: 0 = “not at all,” 4 = “extremely”). A total symptom severity score was obtained by summing participants’ responses to the 20 items. A score of 33 is the clinical cut-off for PTSD. Cronbach’s alpha for the present study was 0.95. *Depression Symptoms* were measured by the Patient Health Questionaire-8 (PHQ-8) (Kroenke et al., 2009). The PHQ-8 is an eight-item, self-administered scale that is based on DSM-IV diagnostic criteria, identical to the PHQ-9, but without the suicide item. Since real-time monitoring of all individuals in this anonymous online survey was not feasible, we used the PHQ-8, choosing to remove the suicide question (Wells et al., 2013). Each item is scored on a four-point scale with responses ranging from “not at all” to “nearly every day.” A PHQ-8 score of ten or more has been found to have 88% sensitivity and 88% specificity for the diagnosis of major depression based on clinical interview. Cronbach’s alpha for the present study was 0.87. *Anxiety Symptoms* were measured by the Generalized Anxiety Disorder-7 (GAD-7) (Spitzer et al., 2006). The response options of the GAD- 7 items are identical to the PHQ-8 and range from 0 to 21, with scores of ≥5, ≥10, and ≥15 representing mild, moderate and severe anxiety symptom levels, respectively. Cronbach’s alpha for the present study was 0.90. *Somatic Symptoms* were measured by the Patient Health Questionaire-15 (PHQ-15) (Kroenke et al., 2002). The PHQ-15 is a fifteen-item scale consisting of 15 somatic symptoms, including ten symptoms of DSM-IV somatization disorder. The PHQ-15 scores somatic symptoms as 0 (not bothered at all), 1 (bothered a little), or 2 (bothered a lot). A total sum of ≥15 indicated high somatic symptom severity based on data from primary care settings. Cronbach’s alpha for the present study was 0.79.

#### Childhood Sexual Assault

The history of Childhood Sexual Abuse was derived using the Child abuse item from the Adverse Childhood Experiences Questionnaire (ACE) (Felitti et al., 1998). The ACE consists of ten items that inquire about different adverse childhood experiences, including abuse, neglect, mental illness or substance abuse by family members, and family dysfunction. Cronbach’s alpha for the present study was 0.76.

#### Minimization

We operationalized minimization using the Centrality of Events Scale-Short Form (Berntsen and Rubin, 2006). This scale has been utilized to measure the integration of trauma into one’s identity. The short form is a 7-item scale that is reliable and valid in trauma populations (α = 0.88), with lower scores indicating less centrality of the trauma. Cronbach’s alpha in the present study was 0.93.

#### Hope and Fulfillment

Hope and fulfillment were measured in this study using the positive psychology instruments, including Post-traumatic Growth, Sense of Coherence (SOC), and Self-Compassion. *Post-traumatic growth* was measured by the Post-traumatic Growth Inventory (PTGI) (Tedeschi and Calhoun, 1996), which is a 21-item self-report instrument used for assessing psychological growth following a traumatic event. The PTGI has five subscales: New Possibilities (e.g., “Established a new path for my life”); Relating to Others (e.g., “A sense of closeness with others”); Personal Strength (e.g., “Knowing I can handle difficulties”); Spiritual Change (e.g., “I have a stronger religious faith”); and Appreciation for Life (“Appreciating each day”). Participants in this study were asked to indicate for each of the statements the degree to which this change occurred in their life since their *most distressing* or *traumatic* unwanted sexual experience as an undergraduate. Scores on the PTGI range from 1 to 126, with higher scores reflecting greater perceived growth. Items on the PTGI range from 1 (“I did not experience this change as a result of my crisis”) to 6 (“I experienced this change to a very great degree as a result of my crisis”). Cronbach’s alpha for the present study was 0.90. One’s sense of coherence was measured with the SOC which is a 13-item scale that examines three dimensions: comprehensibility, manageability, and meaningfulness (Antonovsky and Sagy, 1986; Antonovsky, 1993). The SOC questionnaire has been used in at least 33 languages in 32 countries with at least 15 different versions of the questionnaire. The Cronbach’s alpha values in 127 studies using SOC-13 range from 0.70 to 0.92. Alpha reliability for this study was 0.84. Self-Compassion was measured with the Self-Compassion Scale short form (SCS) (Neff, 2003; Raes et al., 2011). The SCS measures the proposed self-protective aspects of being kind toward oneself in instances of pain or failure; perceiving one’s experiences as part of the broader experience and holding painful thoughts and feelings in balanced awareness. Questions are rated on a Likert scale from 1 (almost never) to 5 (almost always). The six subscales measure an individual’s level of self-kindness, self-judgment, common humanity, isolation, mindfulness, and over-identification. Alpha reliability for this study was 0.75.

### Procedures

This study was approved by the University of Michigan Institutional Review Board (HUM00144780). Study participants were invited to enroll in this study through an email listserve that was sent out by the university sexual assault center, as well as a health research portal designed to connect individuals who have utilized their health care system’s services to potential research opportunities. Eligibility for this study included self-identifying as female, and self-identifying as having had an unwanted sexual encounter while enrolled at a university as an undergraduate (or while an undergraduate who have graduated within 5 years before completion of the survey). Participants entered the online survey following a link, and the opening page was the informed consent document. Completion of the survey served as informed consent. Participants received a $10 gift card upon completion of the 30-minute survey if they provided a mailing address. Participants were offered the option to participate in an additional narrative interview. Participants who expressed interest in the interview portion of the study were contacted by study staff within 2 weeks upon completion of the survey to schedule an appointment for their interview (*n* = 24). At the beginning of the interview, another informed consent form was reviewed, and participants gave signed informed consent for the interview and audiotaping. The narrative interview took about 90 min, and participants were compensated $25 for their time. Digital voice recordings were uploaded to a secure research dropbox. Interviews were transcribed, with the accuracy of the transcription checked and finalized by the interviewer.

### Analysis

Qualitative analysis was conducted using ATLAS.*ti* (Muhr, 2006), and detailed results of this analysis are published elsewhere (Sinko and Saint Arnault, in press). The qualitative analysis was carried out by the LS, and the aim was to identify how contextual and internal processing impacted healing after USE, as well as nature and meaning of healing for the survivors. Transcripts were read and re-read with the central concepts consisting of systematic identification of grounded and axial codes in line-by-line constant comparison (Glaser and Strauss, 1967; Charmaz, 2001). ATLAS.*ti* qualitative software was used for data management and analysis (Muhr, 2006). An audit trail was maintained for scientific rigor.

#### Mixed Method Analysis: Data Transformation

The data transformation technique was used to accomplish mixing of the data sets (Sandelowski, 2000; O’Cathain et al., 2010; Fetters et al., 2013). In order to treat the 50 qualitative codes that emerged from qualitative analysis as quantitative data, we examined the frequency counts for each code, which provides numerical data. However, for this study, there were too many low frequencies variables. Therefore, the first author used qualitative abstraction to group qualitative codes into meaningful, higher-order, experientially-related phenomenon, which we referred to as a “complex.” This abstracting of codes reduced the number of phenomena, and also increased the frequencies for any given phenomena. For example, in the original 50 codes, 15 codes comprised different aspects of the phenomenon of “negative impact” of GBV ranging from mistrust to difficulty setting boundaries, to being unable to identify the impact of the event, with code frequencies ranging from 3 to 10. Grouped, however, the “Negative Impact” complex had a frequency of 213 (see Table 1).

**TABLE 1:** Coding Complexes.

**Complex title (Code frequency)**	**Original qualitative codes**	**Qualitative code definition**
**Contextual dimension**		
Normalization (128)	Normalization of Male Sexual Aggression	Any statement justifying or rationalizing male sexual aggression (i.e., catcalling, coercion) as “normal”
	Observed social beliefs about sexual violence	Statements noting the influence of one’s peer or family beliefs as impacting their own beliefs or decision-making surrounding sexual violence (i.e., rape myths: feeling that the victim is to blame, thinking that rape is equivalent to “bad sex,” thinking that one’s perpetrator is not capable of doing such acts)
	Gender expectations	Expressions of personal or peer beliefs about what is women/men are “expected” to do in sexual scenarios or within society in general
**Internal processing dimension**		
Minimization (63)	Identity: Questioning one’s identity as a survivor	Derived from Identity
	Internal Processing: Lack of clarity	Derived from “labeling clarity”
Negative Impact (213)	Mistrust in the ability to keep oneself safe	Mistrusting one’s abilities of keeping themselves safe related to being emotionally close to one’s perpetrator before or after the USE experience, feeling as though they were unable to “see the signs”
	Comparison to peers	Internally comparing one’s emotions and experiences to their peers, resulting in feelings of inadequacy
	Reclaiming one’s body	Any reference to how one feels about their body post USE. References to body image issues or negative beliefs about one’s own body or feeling unable to connect to one’s body on the body map activity
	Hopelessness	Feeling as though one will never feel better or fear of future consequences related to one’s USE experience
	Feelings of Lack of Belonging	Feeling as though one is isolated or does not fit in
	Disconnection	Any reference to feelings of disconnection or separation from oneself, others, and the world since their USE in their life
	Setting Boundaries	Any reference to an individual communicating their “limitations” or needs post USE with others or intentionally giving themselves space from people or situations that set them back in their healing process
	Internal Processing: Naming the event	Created from splitting the Internal Processing Code
	Regaining control	Attempting to manage one’s body or environment through “taking control”
	“Being Brave” through the discomfort	Being “courageous” or “brave” through being intentional about one’s healing and doing things that make one uncomfortable, yet embracing that feeling and growing from it
	Finding escape	Finding a hobby or place that separates someone emotionally or physically from environments that cause them stress or distress
	Staying active	Intentionally participating in leisure activities that require one to get out of the house and engage their body or mind
	Learning about USE and USE impact	Any reference about the information gathered and learned about USE through the internet or other resources
	Engaging Social Supports	Using one’s social relationships outside of initial disclosure as a means of comfort, support, instrumental help, and/or validation
	Engaging Professional Support	Using professional resources as a means of comfort, support, and/or validation
	Taking care of yourself	Actively engaging in behavior that nurtures oneself despite competing demands (i.e., engaging in therapy, taking medications, eating right, exercising, allowing time for breaks, doing what you love, etc.)
Rumination (239)	Fear of vulnerability	Ignoring or minimizing one’s feelings to others in order to be socially pleasing to be around or because one is afraid of allowing others to see their negative emotions
	Guilt	Any reference to “guilt” or “feeling guilty” as a result of one’s USE impacting one’s life
	Shame	Any reference to “shame” “feeling ashamed” or “feeling embarrassed” as a result of one’s USE impacting one’s life
	Self-blame	Any reference to saying a USE was “their fault” or that they “blamed themselves”
	Anger and Frustration	Feelings of anger and frustration surrounding the injustice of the world, the people in their social world, or their emotions after their USE experience
**Healing dimension**		
Identity (145)	Reclaiming identity	Created from splitting the Rebuilding identity code
	Rebuilding Identity	Expressed beliefs about “survivorship identity” or other specific perceived core attribute one possesses making them “whom they are” in the world as a result of their USE or, in contrast, feeling as though one cannot identify who they are
	Discovering Strength	Discovering or building the belief that one is “strong” or “has strength” after USE experience despite feeling fragile
	Finding your voice	Getting involved in feminist work or being able to stand up for oneself and against social injustice related to USE in general
	Feeling capable	Any reference to feelings of pride, happiness, or hope related to one’s achievements in academics, work, or other important activities
Hope and Fulfillment (148)	Feelings of hope	Feeling optimistic about one’s future and searching for positives within one’s life
	Finding Joy	Discovering what makes you happy and making time that in your life
	Practicing self-acceptance	Being able to “give yourself a break” from the responsibility of USE and working toward loving the person you are while accepting and acknowledging your flaws and struggles
	“Letting go”	Allowing oneself to live in the present and abandon unhealthy thoughts, relationships, feelings, or habits through forgiveness or distance. Statements about moving on from the past or moving forward toward the future
	Helping others	Finding fulfillment through being kind to and aiding others in difficult situations related or unrelated to sexual violence
Engagement (96)	Feelings of belonging	Feeling as though one is cared about and has a place within their social world
	Seeking Universality	Managing loneliness through the meeting, engaging with, and learning about other survivors stories or in contrast, feeling alone in one’s experience
	Building trust	Any reference to the impact of USE on one’s ability to trust others both in friendships and sexually intimate relationships

#### Quantitative Analysis

Quantitative analysis was conducted using SPSS (IBM Corp, 2013). Two quantitative analyses were conducted. First, we compared the mean code frequencies of the qualitative complexes for the eleven women who endorsed a history of childhood sexual assault (CSA/USE) with the 13 women who endorsed unwanted sexual contact (USE alone) using *t*-tests. These analyses of the means of this small subgroup were not intended to represent the population, but rather to help us identify patterns in the qualitative data. The patterns that emerged–that is, differences between the mean frequencies–were used to generate hypotheses. In other words, when we saw differences in the mean code frequencies (*n* = 24), we speculated about whether there would also be differences in the means for the women in the larger sample (*N* = 206). Hypotheses emerged based on significant mean differences for the CSA/USA and USE alone (see Table 2). We then developed a plan to test these hypotheses by operationalizing the phenomenon in the larger survey dataset. Hypothesis 1: Survivors of CSA will have less minimization. We operationalized “minimization” in the larger quantitative dataset as lower Centrality of Trauma Integration scores. Hypothesis 2: Survivors of CSA will have more Negative Impact. We operationalized negative impact as PTSD (PSC5), Depression (PHQ9), Physical Symptoms (PHQ15), and Anxiety (GAD7). Hypothesis 3: Survivors of CSA will have more hope and fulfillment. We operationalized hope and fulfillment as SOC, Post-Traumatic Growth, and Self-Compassion. Finally, we tested these hypotheses using regression analyses.

**TABLE 2:** Significant Qualitative Code complex mean frequency differences between CSA/USE and USE only.

**Qualitative code complex**	**F**	**Significant**	***t***	**df**
Minimization complex	4.265	0.051	0.700	22
Negative Impact complex	4.129	0.054	−1.592	22
Hope and Fulfillment complex	6.755	0.016	−0.607	22

## Results

### Description of Participants

Because of the sampling criteria, all of the participants of the interview had USE on the college campus. The survey respondents included 206 women ranging in age from 18–30 years old (M = 21.9, SD = 2.54). Fifty-four percent of women were current undergraduate students, with 46% of our sample being alumni who graduated a 4-year institution up to 5 years before the survey. Seventy-eight percent of our sample identified as Caucasian, 6% identified as African American or Black, 11% identified as Asian, and 4% identified as a mix of two or more races. Seventy-six percent of our sample identified as heterosexual, 3% of identified as homosexual, 14% identified as bisexual, and 7% identified as “other.” Thirty-one women in the survey sample indicated a history of Childhood Sexual Abuse on the survey.

The interview participants (a nested subsample of the survey sample) included 24 women ages 18–26 were included in the qualitative analysis. Thirteen women were current undergraduate students at a 4-year institution, and eleven women were alumni. Eighteen women identified as Caucasian, three women identified as African American or Black, and three women identified as Asian. In the interview, eleven women self-disclosed a history of childhood abuse, and the remaining women endorsed USE alone.

### Hypothesis Identification for Qualitative Data

In order to develop hypotheses for testing, we used a *t*-test comparison of mean frequencies for the USE/CSA and the USE only groups (see Table 2). Two of the complexes had mean differences approaching significance. Survivors of USE alone had higher Minimization complex frequency means (M = 2.85, SD = 0.90) than survivors of both CSA/USE (M = 2.36, SD = 2.29) (*t* = 0.70, df22, *p* = 0.51). From this trend, we developed the hypotheses that survivors of both USE and CSA would have less minimization. Also, survivors of both USE and CSA had higher Negative Impact complex frequency means (M = 10.8, SD = 6.69) than survivors of USE alone (M = 7.23, SD = 4.27) (*t* = −1.59, *df*22, *p* = 0.54). From this trend, we developed the hypothesis that survivors of both USE and CSA would have higher negative impact scores. Finally, survivors of both USE and CSA had significantly higher Hope and Fulfillment complex frequency means (M = 6.73, SD = 5.31) than survivors of USE alone (M = 5.69, SD = 2.87) (*t* = −0.607, *df*22, *p* = 0.02). From this trend, we developed the hypothesis that survivors of both CSA and USE would have more Hope and Fulfillment.

#### Hypothesis 1: Survivors of Both USE and CSA Will Have Less Minimization Than Survivors of USE Alone

We operationalized minimization as lower Centrality of Trauma Integration scores. This hypothesis was supported in the larger dataset. Survivors of both CSA and USE had a significantly higher mean Centrality Scores (M = 26.8, SD = 5.8) than survivors of USE alone (M = 20.6, SD = 7.8) (*t* = −4.3, *df*191, *p* = 0.00) (see Table 3).

**TABLE 3:** Quantitative scores.

	**USE M (SD)**	**USE/CSA M (SD)**	**Significant**
PTSD	23.36 (16.93)	39.45 (15.95)	0.00
Depression	7.73 (5.25)	12.0 (5.31)	0.00
Anxiety	7.50 (5.29)	11.42 (5.27)	0.00
Somatic	8.33 (5.11)	11.29 (5.16)	0.00
Centrality of Trauma	20.59 (7.81)	26.77 (5.85)	0.00
Post-Traumatic Growth	25.70 (19.43)	33.90 (17.96)	0.04
Self-Compassion	31.59 (6.53)	27.76 (5.74)	0.01
Sense of Coherence	50.15 (11.89)	41.96 (10.89)	0.00

#### Hypothesis 2: Survivors of Both USE and CSA Will Have Higher Negative Impacts Than Survivors of USE Alone

We operationalized negative impact as PTSD (PCL5), Depression (PHQ9), Physical Symptoms (PHQ15), and Anxiety (GAD7). All negative impact scores were significantly higher for the survivors of both CSA and USE. Survivors of both CSA and USE had a significantly higher mean PCL5 score (M = 39.5, SD = 16) than survivors of USE alone (M = 23.4, SD = 17) (*t* = −4.9 (df191), *p* = 0.00). Survivors of both CSA and USE had a significantly higher mean PHQ8 score (M = 12.0, SD = 5.3) than survivors of USE alone (M = 7.7, SD = 5.2) (*t* = −4.1 (df191), *p* = 0.00). Survivors of both CSA and USE had a significantly higher mean GAD7 score (M = 11.4, SD = 5.3) than survivors of USE alone (M = 7.5, SD = 5.2) (*t* = −3.8 (df191), *p* = 0.00). Survivors of both CSA and USE had a significantly higher mean PHQ15 score (M = 11.3, SD = 5.2) than survivors of USE alone (M = 8.3, SD = 5.1) (*t* = −3.0 (df191), *p* = 0.00).

#### Hypothesis 3: Survivors of Both USE and CSA Will Have More Hope and Fulfillment

We operationalized hope and fulfillment as SOC, Post-Traumatic Growth, and Self-Compassion. This hypothesis partially supported. Survivors of both CSA and USE had a significantly higher mean PTG score (M = 33.9, SD = 18) than survivors of USE alone (M = 25.7, SD = 19.4) (*t* = −2.1 (df185), *p* = 0.04). However, survivors of USE alone had significantly higher SOC scores (M = 50.1, SD = 11.9) than survivors of both CSA and USE (*n* = 24) (M = 42.0, SD = 10.9) (*t* = 3.2 (*df*171), *p* = 0.00). Survivors of USE alone also had significantly higher SCS scores (M = 31.6, SD = 6.5) than survivors of both CSA and USE (M = 27.8, SD = 5.7) (*t* = 3.2 (df173), *p* = 0.01).

## Discussion

The finding that those who were sexually attacked as children and again as an adolescent or young adult had a higher negative impact is, heartbreakingly, not a surprise. The advancement of the science in this study was the finding that complex trauma had an impact on other measures as well, including the centrality of impact, physical symptoms (which are rarely examined) and positive health indicators.

Protective psychological mechanisms may be at play when survivors try to cope with these horrific crimes, and the need for these psychological protections is compounded when the victim is close to the perpetrator, such are the cases of incest, child abuse, and date rape. Moreover, the socio-cultural milieu that tolerates, excuses, and normalizes sexual assault makes clear labeling a challenge for survivors (Garcia et al., 2012; Sharp et al., 2017). For this study, we used the centrality of impact as an indicator of minimization, which provided an interesting view into the dynamics of integration of an event into one’s self-view. However, complex trauma recovery is comprised of recovery from multiple trauma experiences, which build up in the hearts and minds of the survivor during critical developmental periods, making a single indicator of trauma integration incomplete. Moreover, overlapping processes of minimization, psychological protection, and the impact of the socio-cultural climate on labeling are not captured in this indicator, substantiating the need for more complex research strategies to inform our therapeutic practices.

One instructive finding in this study was that Post-Traumatic Growth was higher for those survivors of both kinds of trauma history. This finding may be related to the fact that most of the literature on PTG has examined survivors who have experienced one specific type of trauma, and the limited research on those who have had multiple traumatic events, or those who have had single traumatic events, without assessing for additional traumas people may have faced in their lifetime (Shakespeare-Finch and Armstrong, 2010). Our finding is similar to other research which has shown that individuals with complex trauma across the lifespan are more likely to experience post-traumatic growth (Peterson et al., 2008; Shigemoto and Poyrazli, 2013), but is in contrast to others who have found the opposite (Kılıç et al., 2016). Regardless, this finding is hopeful because it seems to reveal some resilience, or perhaps a less studied but important capacity for survivors with a history of multiple traumas to overcome these tragedies. There is no doubt that this is a nascent area in need of research, which could identify areas of empowering interventions that support engagement in psychosocial growth after trauma. Moreover, while hope and fulfillment were high in the interviewed sample, suggesting that there might be some sort of resilience or recovery pathway unique to this group, the quantitative impact seemed to be limited to the PTG domain. Other positive psychology indicators, such as a sense of coherence and self-compassion scores, were lower for those survivors of both kinds of trauma.

### Limitations

This study used innovative techniques to explore relationships among variables that emerged in a qualitative study and could be further tested in a larger sample. While it is interesting to explore these with a nested sample, it is not necessary for the use of this technique. What is necessary is that the samples are related to each other in some way—that is, they are from the sample population and have had similar measurement techniques. Of course, this sample is self-selected, both by taking the survey and then by participating in the interview. Furthermore, they were sampled for a specific university population, so these findings may not be generalizable to other samples of university women, or college-aged women. The survivors in this sample represented a specific American geographical region, a prestigious university setting, and sampled women who volunteered to complete an online survey. The ethnic, racial, and gender expression is not representative of Americans. Moreover, this sample’s experiences are not necessarily generalizable to women from cultures outside the United States. Likewise, these were predominately white heterosexual women, and these relationships might be very different for women representing other identities. Still, we find these trends illuminating and believe that they warrant further exploration of the use of mixed methods in these populations. Further research on the trends revealed in this study represent an important beginning step to understand what healing experiences might be for this vulnerable subsample of women.

### Future Directions

This study employs a novel approach to explore healing experiences and needs for a sample of college women. We believe that this approach has value because it allows us to listen deeply to the experiences and perspectives of assault survivors, and then use this data to explore trends in a larger dataset in the same study. At a time when societal problems such as sexual violence are at public health crisis levels, we must speed up our research and employ new and creative methods to understand these complex phenomenon. Moreover, we encourage clinicians to recognize the strengths and healing journeys of the subgroups within their clientele, allowing us to develop appropriate and meaningful treatment strategies.

## Data Availability

The datasets generated for this study are available on request to the corresponding author.

## Ethics Statement

This study was approved by the University of Michigan Institutional Review Board (HUM00144780).

## Author Contributions

All authors listed have made a substantial, direct and intellectual contribution to the work, and approved it for publication.

## Conflict of Interest Statement

The authors declare that the research was conducted in the absence of any commercial or financial relationships that could be construed as a potential conflict of interest.
